# Unstable cardiac injury complicated with septic shock—a challenge

**DOI:** 10.1186/s41038-016-0035-y

**Published:** 2016-04-08

**Authors:** Neha Garg, Kapil Dev Soni, Richa Aggarwal

**Affiliations:** 1 Department of Anaesthesia and Intensive Care, All India Institute of Medical Sciences, Ansari Nagar East, Gautam Nagar, New Delhi, 110029 India; 2 Division of Critical & Intensive care, All India Institute of Medical Sciences, Ansari Nagar East, Gautam Nagar, New Delhi, 110029 India

**Keywords:** Blunt cardiac injury, Sepsis, Myocardial depression, Troponin

## Abstract

**Background:**

Road traffic accident accounts for 70 % to 80 % of the blunt cardiac injury. The true incidence varies in the literature due to non-uniform criteria for diagnosis.

**Case Presentation:**

Here, we describe the case of a young male presenting after blunt chest injury and hemodynamic instability. Initially, the patient had frequent episodes of arrhythmias and hypotension due to cardiac injury per se. However, he was stabilized by day 2. Subsequently, patient developed cellulitis followed by septic shock and succumbed to cellulitis on day 5 of injury.

**Conclusion:**

Sepsis is difficult to be diagnosed and treated in the presence of cardiac injury. Myocardial depression has been found in sepsis, which contributes as an added comorbidity in an already compromised heart function. Sepsis also interferes with the diagnosis and follow-up of progress of blunt cardiac injury.

## Background

Road traffic accident accounts for 70 % to 80 % of the blunt cardiac injury [1]. But due to non-availability of fixed criteria for its diagnosis, its exact incidence cannot be quantified [2]. It is reported to range between 8 % and 76 % [3]. Sepsis superimposed on blunt cardiac injury not only makes diagnosis difficult but also worsens prognosis.

## Case presentation

A 25-year-old male patient presented with a history of fall of a large stone on chest at a construction site followed by loss of consciousness and fall on back. On initial presentation, blood pressure (BP) was 60/36 mmHg and pulse was 184 beats/min. He was immediately intubated and resuscitated with fluids and inotropes. The patient responded by stabilising BP to 110/60 mmHg. Bilateral intercostal drains were put. Contrast enhanced CT (CECT) chest showed mild pericardial fluid, fractured third rib, and bilateral hemopneumothorax. The patient also had grade 3 liver injury. The patient was shifted to the intensive care unit (ICU) for further management.

**Fig. 1:**
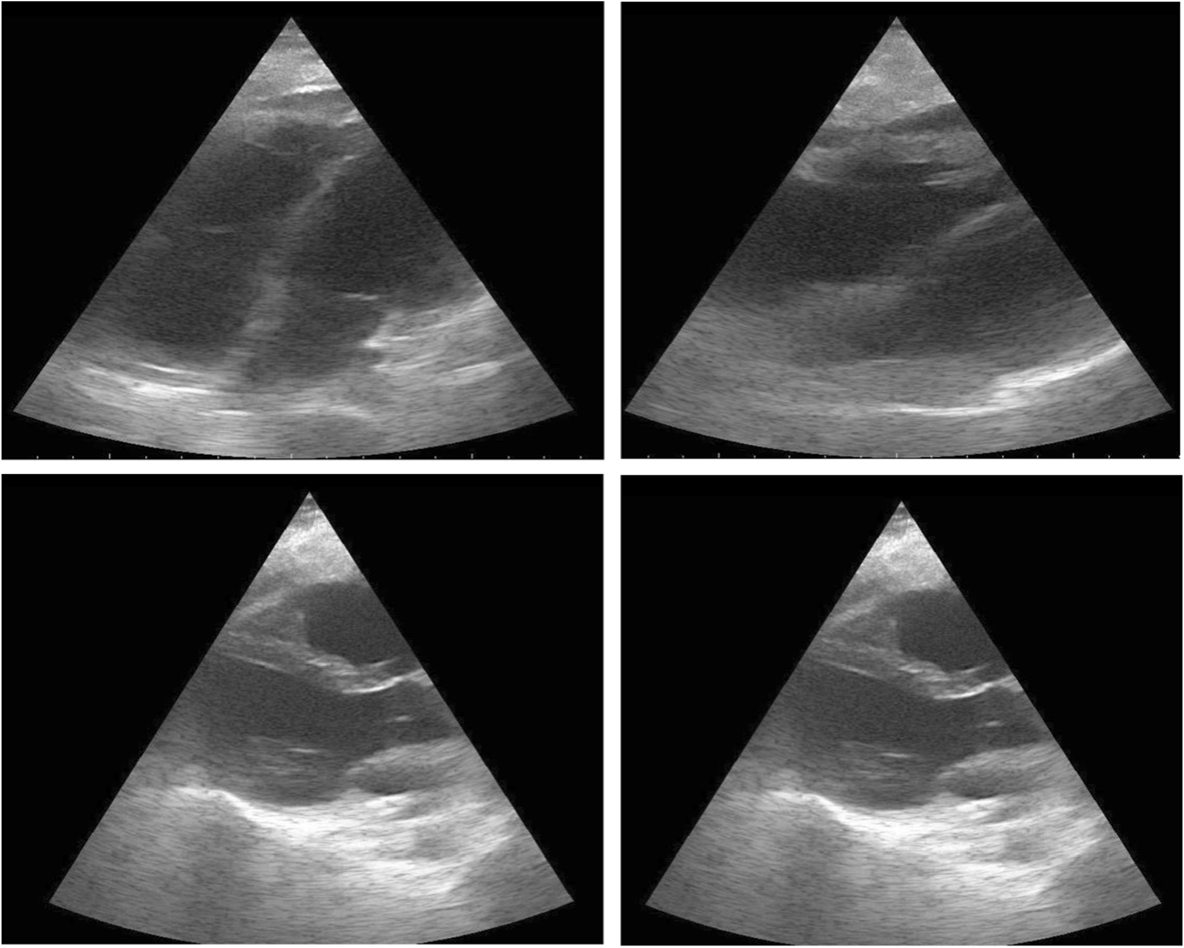
Echocardiogram. Showing areas of regional wall motion abnormalities

**Fig. 2:**
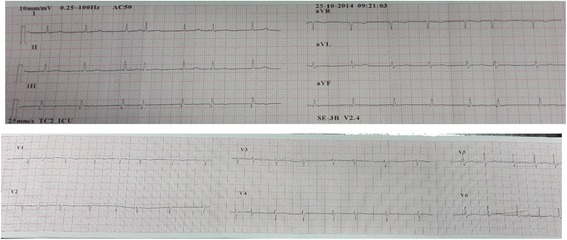
Electrocardiogram. Showing the presence of atrial fibrillation

On arrival at the ICU, there was a sudden drop in BP with new onset arrhythmias. Electrical alternans was noted on electrocardiogram as well. 2D echocardiogram (Fig. 1) demonstrated mild to moderate pericardial fluid, regional wall motion abnormality, and low ejection fraction. Therefore, urgent pericardiocentesis was done, and BP improved marginally afterwards. The patient developed repeated episodes of atrial fibrillation (Fig. 2) and ventricular tachycardia with hemodynamic instability requiring frequent defibrillations. The patient became hemodynamically stable with control of arrhythmias within 2 days. CPK MB, troponin T, and troponin I were found to be positive. Repeat echocardiography showed a decreased ejection fraction of 25 % to 30 %.

On the third day after ICU stay, the patient developed cellulitis on the back and half of thigh with high grade fever episodes and new onset hemodynamic instability. Immediately, cultures (blood, urine, and tracheal) were sent and antibiotics were escalated. However, no organism could be isolated probably because antibiotics had been initiated empirically. Meanwhile, crystalloids were administered cautiously under echo guidance. However, the patient continued to deteriorate and succumbed to septic shock on the fifth day of injury.

Most patients with cardiac injury have fatal outcome before reaching the hospital. Penetrating cardiac injury, requirement of inotropes, and the presence of new onset arrhythmias are associated with worse survival. However, sepsis as a worse prognostic factor has not been reported previously. Here, we present a case where sepsis complicated the blunt cardiac injury and leads to fatal outcome.

Wijngaarden and associates et al. in their 10-year institutional review noted a 14 % mortality in patients with blunt cardiac trauma which increased to 58 % in patients requiring inotropic support [1]. The presence of arrhythmias decreases the survival to only 12 % to 13 % [4].

Blunt cardiac injury manifests with wide array of signs and symptoms varying from benign ectopic beats to fatal cardiac arrhythmias, shock leading to sudden collapse and death. It may also result in the rupture of the myocardium, pericardium, or valve assembly presenting with gross hemodynamic instability. The right ventricle is the most commonly to be injured portion of heart. However, subtle signs such as flail chest, ecchymosis, and sternal fractures may be the only presentation. Combined use of ECG and troponin I has been found to be one hundred percent sensitive for detection of clinically significant blunt chest trauma [5] (defined as cardiogenic shock, dysrhythmias requiring treatment, or structural cardiac abnormalities) [6]. A normal screening ECG rules out any significant cardiac injury and predicts a benign course [7]. However, troponin has also been found to be elevated in patients with septic shock making it unreliable for diagnosing in septic patients [8]. Echocardiography is an excellent tool for diagnosing and monitoring patient with blunt cardiac injury [9]. However, its interpretation becomes confounded by the presence of sepsis (Table 1).

**Table 1:** Inflammatory variables and tissue perfusion variables

Inflammatory variables^a^	Fever >38 °C, TLC—21,000
	Heart rate greater than 100
Tissue perfusion variables	BP 60/40 on receiving. ^#^Improving initially but increased ionotropic support since 3rd day.
	Urine output decreased on 3rd day and became oliguric by 4th day

^a^The above variables can be due to trauma as well as systemic inflammatory response syndrome, the precursor of sepsis

^#^Sepsis added to it will cause hemodynamic instability which was already present in this patient thus confounding the presence of sepsis

*TLC* Total lymphocyte count; *BP* Blood pressure

Sepsis added to cardiac injury may lead to worse prognosis. Myocardial depression, in the form of biventricular dilation and decreased ejection fraction, has been demonstrated in most patients with septic shock [10]. This further complicates the management of patients with cardiac injury by acting as a “second hit” to the injured heart. This depression is however reversible over 7 to 10 days in survivors. End-diastolic dysfunction and end-systolic dysfunction are also found contributory to poor prognosis [11].

## Conclusions

Early diagnosis and management of sepsis becomes the utmost priority in the presence of blunt cardiac injury. Signs and symptoms are often masked by cardiac injury leading to delayed diagnosis of sepsis. High index of suspicion and close monitoring is the key to identify the onset of severe sepsis. Probably an early initiation of antibiotics may help treat sepsis when it is masked by the presence of blunt cardiac injury. However, no recommendation can be made on the basis of a single case report, and the use of broad-spectrum antibiotics in blunt chest trauma in the absence of any definitive diagnosis may in fact lead to indiscriminate use of antibiotics and development of further resistant strains. Further studies are thus needed to identify markers of early sepsis in patients of blunt cardiac injury rather than blind antibiotic coverage in this group.

## Consent

Written informed consent was obtained from the patient’s parents for the publication of this report and the accompanying images.

## Abbreviations

%: percent
CECT: contrast enhanced CT
ECG: electrocardiogram
ICU: intensive care unit
